# Predicting low cognitive ability at age 5 years using perinatal data and machine learning

**DOI:** 10.1038/s41390-023-02914-6

**Published:** 2024-01-04

**Authors:** Andrea K. Bowe, Gordon Lightbody, Daragh S. O’Boyle, Anthony Staines, Deirdre M. Murray

**Affiliations:** 1 INFANT Research Centre, University College Cork, Cork, Ireland; 2 Department of Electrical and Electronic Engineering, University College Cork, Cork, Ireland; 3 School of Nursing, Psychotherapy, and Community Health, Dublin City University, Dublin, Ireland; 4 Department of Paediatrics, Cork University Hospital, Cork, Ireland

## Abstract

**Background:**

There are no early, accurate, scalable methods for identifying infants at high risk of poor cognitive outcomes in childhood. We aim to develop an explainable predictive model, using machine learning and population-based cohort data, for this purpose.

**Methods:**

Data were from 8858 participants in the Growing Up in Ireland cohort, a nationally representative study of infants and their primary caregivers (PCGs). Maternal, infant, and socioeconomic characteristics were collected at 9-months and cognitive ability measured at age 5 years. Data preprocessing, synthetic minority oversampling, and feature selection were performed prior to training a variety of machine learning models using ten-fold cross validated grid search to tune hyperparameters. Final models were tested on an unseen test set.

**Results:**

A random forest (RF) model containing 15 participant-reported features in the first year of infant life, achieved an area under the receiver operating characteristic curve (AUROC) of 0.77 for predicting low cognitive ability at age 5. This model could detect 72% of infants with low cognitive ability, with a specificity of 66%.

**Conclusions:**

Model performance would need to be improved before consideration as a population-level screening tool. However, this is a first step towards early, individual, risk stratification to allow targeted childhood screening.

**Impact:**

- This study is among the first to investigate whether machine learning methods can be used at a population-level to predict which infants are at high risk of low cognitive ability in childhood.
- A random forest model using 15 features which could be easily collected in the perinatal period achieved an AUROC of 0.77 for predicting low cognitive ability.
- Improved predictive performance would be required to implement this model at a population level but this may be a first step towards early, individual, risk stratification.

## Introduction

Early life is a unique period where the developing brain has great plasticity and huge potential for adaptability.^1^ There is consensus agreement that individual interventions to improve cognitive development should be initiated early.^2,3^ A failure to achieve early foundational cognitive skills may result in a permanent loss of opportunity to reach full academic potential.^4^ This, in turn, may adversely affect outcomes throughout the life course including educational attainment,^5^ mental health,^6^ social mobility,^7^ financial well-being,^8^ and physical health.^9^

Internationally, many countries rely on universal screening programmes to identify children who may benefit from early intervention. The majority of developmental screening assessments are based on the presence of a delay in developmental milestones.^10^ A limitation of this approach is that opportunities for intervention in the period of optimal neuroplasticity are lost, and intervention begins when an infant is already substantially behind their typically developing peers. There is increasing evidence that early pre-emptive interventions, initiated prior to overt signs of delay or difficulty, can alter neurodevelopmental outcomes.^11–13^ The challenge we face is predicting at an individual level, using accurate and scalable methods, who the highest risk infants are.

In the United States, the population-based early intervention programmes Head Start and Early Head Start, base eligibility primarily on a family income at or below the poverty level.^14^ Adverse socioeconomic conditions are among the strongest predictors of poor cognitive outcomes in childhood, but there are other important psychosocial, biological, genetic, and environmental influences, which often have complex and interactive relationships both with each other and with cognitive outcomes.^15,16^ There is now increasing potential to statistically model complex interactive relationships using machine learning techniques.^17,18^ This has not been sufficiently explored for prediction of poor cognitive outcomes in childhood at a population level.^19^

In this study we aim to develop an explainable predictive algorithm, using population-based cohort data, for identifying infants at risk of low cognitive ability (LCA) at school-age. The objectives of the study are to train a variety of machine learning models to predict LCA at age 5; to test these models on an independent unseen test-set; to compare model performance using a range of measures; and to identify the most important features for prediction.

## Methods

### Data

Data are from the Growing Up in Ireland (GUI) Infant Cohort, a nationally representative survey of infants and their primary caregivers (PCG). Wave 1 of data collection commenced in 2008 at infant age 9-months, Wave 2 occurred at age 3 years, and Wave 3 at 5 years. The sample was drawn from the National Child Benefits Register, a universal welfare entitlement in the Republic of Ireland. It was selected on a systematic basis with a random start and constant sampling fraction, and was pre-stratified by marital status, county of residence, nationality, and number of children in the family. There were 11,134 families who participated at Wave 1, of whom 9001 completed Wave 3, representing 80.8% of the original sample. Full details of the sample design, response, and survey instruments are available.^20^ Eligible for inclusion in this study were the 8858 infants who completed cognitive assessments at age 5 and their PCGs, who in 99.7% of cases were the infant’s mother. A flow chart of the study population is contained in Supplementary Material Fig. S1.

### Outcome

Cognitive ability at age 5 years was directly assessed using two core subtests of the British Ability Scales (BAS) Early Years Battery Second Edition, administered in the child’s home by a trained interviewer. The BAS consists of a battery of individually administered subtests (detailed in Supplementary Material Table S1) and has demonstrated construct validity as a measure of cognitive ability and high test-retest reliability.^21^ To minimise participant burden in the GUI study, there was an upper limit of 90 min contact time in the home. Therefore, it was not feasible to administer the full battery of tests, and two core subtests which most closely align with measures of crystallised and fluid cognitive ability were chosen.^22^

The Naming Vocabulary test measures verbal ability in the English language and consists of the child naming everyday items displayed from a picture book. The Picture Similarities test measures non-verbal ability and consists of the child being shown four pictures and requested to match a fifth picture, based on a shared characteristic or construct. A standardised score for each scale is provided in the dataset and is adjusted for both item difficulty and age (within a 3 month age band).

Multiple BAS subtest scores can be combined with summation to produce a General Conceptual Ability Score. To produce composite scores using fewer subtests principal components analysis (PCA) was used, as in previous research.^23^ PCA of the two BAS subtests confirmed the presence of a general underlying cognitive ability factor. Principal component 1 (PC1) accounted for 64% of the total variance among the subtests. The Pearson correlation between this factor and the observed variable was 0.80 for Picture Similarities and 0.80 for Naming Vocabulary subtests. PC1 was then standardised to produce a general cognitive ability (GCA) score with a mean of 100 and a standard deviation (sd) of 15.

There is no consensus agreement on a cut-off that defines LCA in childhood. The International Classification of Disease 11th Revision (ICD-11) use a standardised test score that is ≥2 standard deviations (SD) below the mean to define a disorder of intellectual development, while other research in this area has used cut-offs of ≥1 or 1.5 SDs below the mean.^16,24,25^ As this study was exploratory in nature, we examined both a 1 and 1.5 SD cut-off. For clarity of presentation the 1.5 SD cut off is used in the main study and children scoring below this cut-off are referred to as having low cognitive ability (LCA). The results using a 1 SD cut off are included in online-only material and this is referred to as below average cognitive ability (BACA).

### Data preparation

No feature had more than 12% missing values. Missing values were imputed using the ‘missForest’ package, a random forest imputation method.^26^ The post-imputation dataset was stratified by the outcome and then randomly split into a training set, containing 70% (*n* = 6202) of participants, and a testing set, containing 30% (*n* = 2656).

### Feature selection

The GUI dataset contains more than 600 variables measured at wave 1 (9-months) across domains of health, education, cognitive development, social class, and neighbourhood characteristics. The framework used in the selection of relevant features is shown in Fig. S2. These features capture pregnancy and birth, maternal and infant characteristics, the socioeconomic circumstances of family, and the infant’s early environment. Only features based on information that could be easily obtained at a population level without the need for invasive testing were eligible for inclusion. Features which could be collected in the perinatal period were preferable to enable prediction soon after birth. It is well established that the early learning environment is very important in cognitive development, and a set of features intended to measure this were included.^27^ Previous published work by our group has focussed on feature selection to predict low IQ in a separate cohort and this was also considered.^28^ All features considered are described in Supplementary Material Table S2.

To remove redundant features, Pearson correlation coefficients were calculated and plotted. Features with a correlation greater than 0.6 were examined for redundancy. In choosing which features to remove, the potential timing of collection, objectivity, and clinical opinion were considered. Three feature sets were then created (Table S3). Set 1 contained the features identified as most important in our previous work.^28^ Set 2 contained only features that could potentially be collected in the perinatal period. Set 3 included those representing the child’s early environment that would require later measurement. Recursive feature elimination (RFE), with five-fold cross validation repeated five times, was performed for each feature set to determine the optimal combination for the final models. The features included in the final models are indicated by an asterisk in Table S3.

### Modelling

The dataset was imbalanced with regard to the outcome of interest. Training a model on class imbalanced data risks producing a model bias in favour of the majority class, with poor predictive performance for the minority class.^29^ To address this Synthetic Minority Oversampling Technique (SMOTE) was applied to the training set only.^30^ Unlike other oversampling techniques which simply duplicate minority class cases, SMOTE utilises an over-sampling approach where the minority class is over-sampled by creating synthetic examples.^29,30^ Random forest (RF), logistic regression (LR), and support vector machine (SVM) algorithms were trained and optimal hyperparameters selected using the rebalanced training dataset and ten-fold cross validated grid search.

### Evaluation

**Fig. 1:**
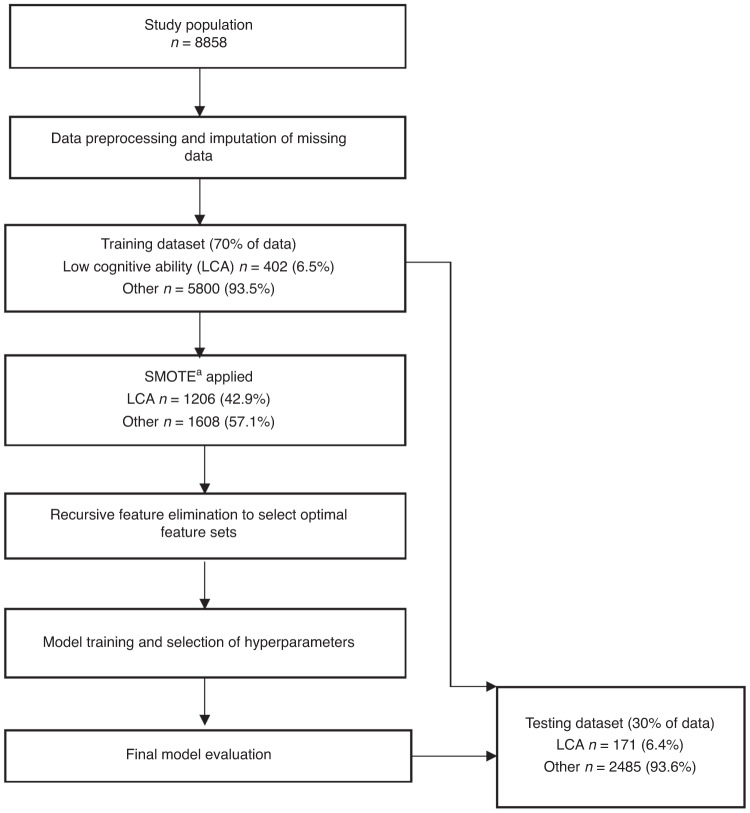
Overview of modelling process. ^a^Synthetic minority oversampling technique.

To select the most appropriate machine learning algorithm, accuracy across ten-fold cross-validation was compared. After selection of the algorithm, the final models were tested on an independent unseen test set. Area under the receiver operating curve (AUROC) was used to evaluate overall model performance. Explanation of performance metrics is provided in online-only material. A summary of the modelling process is contained in Fig. 1.

### Explainability

Feature importance plots were created using the permutation method in the ‘vip’ package.^31^ First, baseline model performance is measured using a measure set by the user (in this study—AUROC). The feature of interest is then randomly shuffled and model performance is measured again. The difference between the two measures is used as a measure of feature importance. For each feature shuffling was simulated ten times and importance was averaged across the simulations. It would be expected that randomly shuffling the values of an important feature would degrade model performance.^31^

The relationships between the most important features and the outcome were examined using partial dependence plots (PDPs), which provide a visualisation of the relationship between a feature and the response while accounting for the average effect of the other features in the model.^32^ A lower value on the *y*-axis of the PDP suggests that the positive class (low cognitive ability) is less likely at that value of the feature on *x*-axis according to the model.

## Results

### Characteristics of study population

There were 8858 infants included, of whom *n* = 573 (6.5%) had LCA. A summary of characteristics are described in Table 1, with a complete description provided in Table S4. The LCA group, which was comprised of 60.6% boys, had a lower mean maternal age (30.6 vs. 32.1 years, *p* < 0.001), a higher proportion of mothers who smoked (30.4% vs. 22.4%, *p* < 0.001), and a lower proportion of mothers reporting English as their native language (53.9% vs. 86.9%, *p* < 0.001). In the LCA group, 43.8% of mothers reported their highest education level as being secondary or primary only, compared with 27.5% of those without LCA. There were significant differences between the groups with regard to all socioeconomic characteristics. The LCA group had a lower median family income (€31,200 vs. €48,000, *p* < 0.001), a lower proportion of families living in owner occupied accommodation (38.9% vs. 73.5%, *p* < 0.001), and a higher proportion in the lower social classes.

**Table 1:** Pregnancy, maternal, infant, socioeconomic and early environmental characteristics of study population.

Characteristics	Total	Low *n* = 573 (6.5%)	Other *n* = 8285 (93.5%)	*p* value
Pregnancy and birth				
Mode of delivery				
Normal vaginal	5239 (59.1)	367 (64.0)	4875 (58.8)	
Forceps/suction assisted	1299 (14.7)	60 (10.5)	1238 (14.9)	
Planned c-section	1151 (13.0)	61 (10.6)	1090 (13.2)	
Emergency c-section	1169 (13.2)	85 (14.8)	1082 (13.1)	0.003^b^
Admission to NICU/SCBU	1217 (13.7)	88 (15.4)	1129 (13.6)	0.271^b^
Gestational age—mean (sd)	39.5 (2.1)	39.3 (2.4)	39.5 (2.0)	0.035^c^
Singleton pregnancy	8546 (96.5)	290 (92.9)	7995 (96.5)	0.075^b^
Birthweight				
<1500 g	67 (0.8)	6 (1.0)	61 (0.7)	
1500 g–2500 g	401 (4.5)	38 (6.6)	362 (4.4)	
2500 g–4500 g	8178 (92.3)	509 (88.8)	7670 (92.6)	
>4500 g	212 (2.4)	20 (3.5)	192 (2.3)	0.0138^e^
Breastfed on discharge—yes	4131 (46.6)	300 (52.4)	4427 (53.4)	0.648^b^
Smoker in hsd^a^ during pregnancy—yes	2801 (31.6)	225 (39.3)	2576 (31.1)	<0.001^b^
Maternal				
Age—mean (sd)	32.0 (5.2)	30.6 (5.7)	32.1 (5.1)	<0.001^c^
BMI—mean (sd)	25.5 (4.7)	25.9 (5.0)	25.5 (4.7)	0.072^c^
Self-rated health				
Excellent	2798 (31.6)	159 (27.7)	2639 (31.9)	
Very good	3505 (39.6)	223 (38.9)	3282 (39.6)	
Good	2020 (22.8)	150 (26.2)	1870 (22.6)	
Fair	475 (5.4)	35 (6.1)	440 (5.3)	
Poor	60 (0.6)	6 (1.0)	54 (0.7)	0.097^b^
Current smoker—yes	2028 (22.9)	174 (30.4)	1854 (22.4)	<0.001^b^
Alcohol intake				
Never	1457 (16.4)	192 (33.5)	1265 (15.3)	
<1/month	2409 (27.2)	178 (31.1)	2231 (26.9)	
1–2/month	2398 (27.1)	119 (20.8)	2279 (27.5)	
1–2/week	2176 (24.6)	73 (12.7)	2103 (25.4)	
3–4/week	353 (4.0)	10 (1.7)	343 (4.1)	
5–6/week	49 (0.6)	<5	48 (0.6)	
Daily	16 (0.2)	<5	16 (0.2)	<0.001^e^
Maternal depression—median (IQR)	1 (3)	1 (3)	1 (3)	0.0384^d^
Chronic illness—yes	1019 (11.5)	55 (9.6)	964 (11.6)	0.159^b^
Highest level of education				
Primary or less	186 (2.1)	43 (7.5)	143 (1.7)	
Lower secondary	739 (8.3)	77 (13.4)	662 (8.0)	
Upper secondary	1608 (18.2)	131 (22.9)	1477 (17.8)	
Technical/vocational	1194 (13.5)	114 (19.9)	1080 (13.0)	
Non degree	1787 (20.2)	74 (12.9)	1713 (20.7)	
Degree or equivalent	1617 (18.3)	68 (11.9)	1549 (18.7)	
Post degree cert/diploma	1047 (11.8)	36 (6.3)	1011 (12.2)	
Post degree masters or PhD	680 (7.7)	30 (5.2)	650 (7.8)	<0.001^b^
Infant				
Infant gender—male	4479 (50.6)	347 (60.6)	4132 (49.9)	<0.001^b^
Socioeconomic				
Household income—median (IQR)	48,000 (33,600)	31,200 (24,963)	48,000 (33,330)	<0.001^d^
Household social class				
Professional workers	1711 (19.3)	52 (9.1)	1659 (20.0)	
Managerial and technical	2863 (32.3)	109 (19.0)	2754 (33.2)	
Non-manual	1472 (16.6)	85 (14.8)	1387 (16.7)	
Skilled manual	1168 (13.2)	122 (21.3)	1046 (12.6)	
Semi-skilled	638 (7.2)	72 (12.6)	566 (6.8)	
Unskilled	119 (1.3)	15 (2.6)	104 (1.3)	
All others gainfully occupied	37 (0.4)	8 (1.4)	29 (0.4)	
Never worked	850 (9.6)	110 (19.2)	740 (8.9)	<0.001^b^
Accommodation occupancy				
Owner occupied	6315 (71.3)	223 (38.9)	6091 (73.5)	
Local authority housing	623 (7.0)	85 (14.8)	538 (6.5)	
Private rental	1622 (18.3)	249 (43.5)	1374 (16.6)	
Living with parents	146 (1.6)	8 (1.4)	138 (1.7)	
Occupied rent free	152 (1.7)	8 (1.4)	144 (1.7)	<0.001^b^
Number of bedrooms—mean (sd)	3.5 (0.9)	3.1 (0.9)	3.5 (0.9)	<0.001^c^
Connectedness in community				
Strongly agree	3777 (42.6)	186 (32.5)	3591 (43.3)	
Agree	4292 (48.5)	320 (55.8)	3972 (47.9)	
Disagree	618 (7.0)	54 (9.4)	564 (6.8)	
Strongly disagree	171 (1.9)	13 (2.3)	158 (1.9)	<0.001^b^
Early environment				
Siblings—yes	5410 (61.1)	355 (62.0)	5055 (61.0)	0.687^b^
Books in home				
None	29 (0.3)	6 (1.0)	23 (0.3)	
<10	585 (6.6)	111 (19.4)	474 (5.7)	
10–20	1626 (18.4)	169 (29.5)	1457 (17.6)	
21–30	1562 (17.6)	105 (18.3)	1457 (17.6)	
>30	5056 (57.1)	182 (31.8)	4874 (58.8)	<0.001^b^
Hours on learning activities—mean (sd)	34.2 (8.5)	31.8 (9.9)	34.4 (8.3)	<0.001^c^
Level of support				
Get enough	6446 (72.8)	344 (60.0)	6102 (73.7)	
Don’t get enough	856 (9.7)	49 (8.6)	807 (9.7)	
Don’t get any	482 (5.4)	36 (6.3)	446 (5.4)	
Don’t need any	458 (5.2)	51 (8.9)	407 (4.9)	
Family not in country	616 (7.0)	93 (16.2)	523 (6.3)	<0.001^b^
English maternal native language—yes	7512 (84.8)	309 (53.9)	7203 (86.9)	<0.001^b^
Partner in household	7929 (89.5)	491 (85.7)	7438 (89.8)	0.003^b^

^a^Household.

^b^Pearson’s chi-squared test.

^c^Welch two sample *t*-test.

^d^Wilcoxon rank sum test.

^e^Fisher’s exact test (*p* value simulated where cell number too small).

### Rebalanced training dataset

In the study dataset, *n* = 573/8858 (6.5%) children had LCA. As detailed in Fig. 1, in the training dataset, which contained 70% of participants, *n* = 402/6202 had LCA. After application of SMOTE, the rebalanced training dataset consisted of *n* = 1206 (42.9%) children with LCA and *n* = 1608 (57.1%) without. This rebalanced dataset was used to train the models. The testing dataset was not rebalanced and contained the original 30% of participants, of whom *n* = 171/2656 (6.5%) had LCA. This was the dataset used to evaluate the models.

### Feature and algorithm selection

Following RFE 8 features were retained for Model 1, 15 for Model 2 and 23 features for Model 3. As shown in Table S5, the random forest algorithm was the best performing algorithm for all three models, in repeated ten-fold cross validation, and was selected for the final models.

### Final model evaluation

The independent test set contained *n* = 2656 participants, of whom *n* = 171 had LCA. Models 2 and 3 achieved the highest AUROC of 0.77 and 0.78 respectively (Fig. S3). At a decision threshold of 0.5 the models correctly predicted the cognitive outcome of 87% of participants at age 5 (Table 2). The odds of having low cognitive ability were 5.9 times higher in the group with a ‘low’ prediction. Model 2 was deemed to be the best performing model overall, achieving similar performance to Model 3 but using only 15 features, all of which had potential to be collected in the perinatal period. To further explore its performance, the decision threshold was altered in increments of 0.5 and the corresponding sensitivities, specificities, positive and negative predictive values were examined using the independent test set. The optimal threshold of 0.28, using Youden’s index, yielded a sensitivity of 72% with a specificity of 66% (Table S6).

**Table 2:** Performance metrics of final models tested on independent test set.

	Model 1 (8 features)^c^	Model 2 (15 features)^d^	Model 3 (23 features)^e^
Accuracy^a^	0.86 (0.85–0.88)	0.87 (0.85–0.88)	0.87 (0.85–0.88)
Sensitivity^a^	0.25	0.40	0.43
Specificity^a^	0.91	0.90	0.89
Positive predictive value^a^	0.16	0.22	0.22
Negative predictive value^a^	0.95	0.96	0.96
AUROC^b^ (95% confidence interval)	0.69 (0.65–0.73)	0.77 (0.73–0.80)	0.78 (0.74–0.81)

^a^Calculated at a decision threshold of 0.5.

^b^Area under receiver operating characteristic curve.

^c^Features—Household social class, household equivalised income, PCG age, gestational age, PCG BMI, PCG alcohol intake, maternal depression score, PCG highest education.

^d^Features—Household social class, household equivalised income, PCG age, gestational age, PCG BMI, PCG alcohol intake, maternal depression score, PCG highest education, number bedrooms in home, English native language of PCG, birthweight category, accommodation ownership, number in household smoking during pregnancy, community connectedness, PCG self-rated health.

^e^Household social class, household equivalised income, PCG age, gestational age, PCG BMI, PCG alcohol intake, maternal depression score, PCG highest education, number bedrooms in home, English native language of PCG, birthweight category, accommodation ownership, number in household smoking during pregnancy, community connectedness, PCG stress score, hours spent on learning activities, books in the home, hours of sleep for PCG, quality of attachment score, PCG employment status, verbal interaction between PCG and baby, level of support received by PCG, gestational diabetes diet.

A worked example of how the model, at a decision threshold of 0.5, would function in the real world is provided in Table S7. In 2021 there were almost 60,000 births in Ireland. Assuming a prevalence of 6.5%, 3900 infants will have LCA at age 5, of whom 1560 would be detected in infancy (sensitivity 0.40). Of the 56,100 infants with average or above cognitive ability at age 5, 50,490 would be correctly identified as not at risk (specificity 0.90). There would be 5610 false positives and 2340 false negatives.

The modelling and results for predicting LCA using an alternative cut off of a GCA score more than 1 SD below the mean are shown in Supplementary Material Fig. S4 and Tables S8 and S9.

### Explainability

**Fig. 2:**
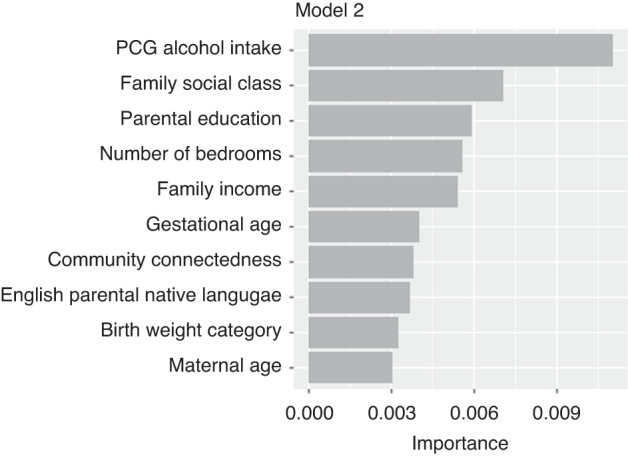
Ten most important features in random forest model 2. This figure plots the ten most important features used in random forest model 2 for predicting low cognitive ability at age 5.

**Fig. 3:**
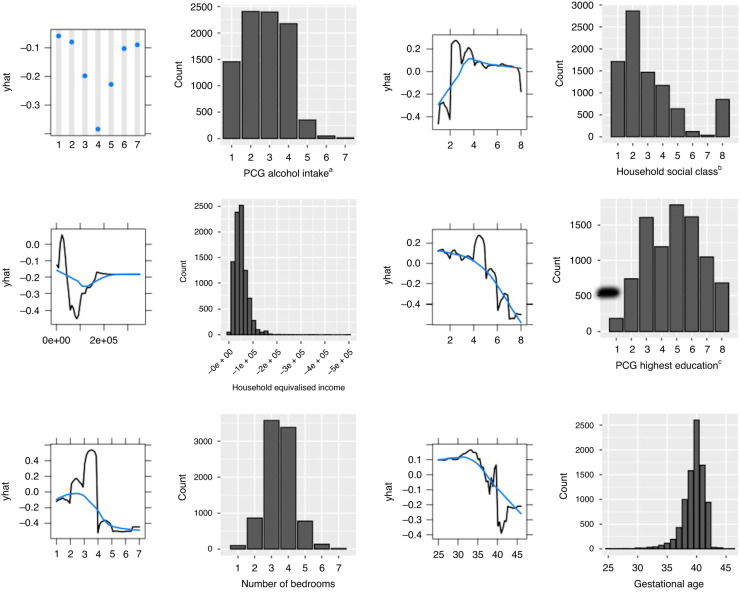
Partial dependence plots and histograms examining the relationship between the six most important features in Random Forest Model 2 and low cognitive ability. Shown in this figure are partial dependence plots (PDPs) for the six most important features in Model 2. Each PDP is accompanied by a histogram showing the distribution of the feature in the original unbalanced dataset. The PDP shows the cumulative effect (*y*-axis), according to our model, of each predictor’s individual values (*x*-axis) on classification of the outcome. The absolute values on the *y*-axis are affected by class imbalance. The overall shape and directionality are important for interpretation. The effect of features of class factor are shown in dots (e.g. PCG alcohol intake) and the effect of features of class numeric are shown with a black line. The blue line represents a locally estimated scatterplot smoothing line (LOESS) which attempts to capture the general relationship while reducing noise. For example, in the PDP plot for household social class we can see that the highest social class categories predict low cognitive ability less strongly than the lower social class categories. ^a^Primary caregiver alcohol intake categories: 1—Never, 2—Less than once per month, 3—1–2 times per month, 4—1–2 times per week, 5—3–4 times per week, 6—5–6 times per week, 7—Everyday. ^b^Household social class categories: 1—Professional workers, 2—Managerial and technical, 3—Non-manual, 4—Skilled manual, 5—Semi-skilled, 6—Unskilled, 7—All others gainfully employed, 8—Never worked at all—no class. ^c^Primary caregiver highest education categories: 1—Primary or less, 2—Lower secondary, 3—Upper secondary, 4—Technical or vocational qualification, 5—Non-Degree, 6—Degree or equivalent, 7—Post degree professional qualification or certificate or diploma, 8—Postgraduate masters or PhD.

The five most important features in Model 2 were PCG alcohol intake (measured when infant was 9-months old), household social class, PCG highest education, number of bedrooms in the home, and household equivalised income (Fig. 2). PDPs are shown in Fig. 3, alongside a histogram showing the distribution of each feature in the dataset.

The five most important features were all markers of socioeconomic status (SES). To determine whether any of these markers of SES could achieve similar predictive performance alone, five separate random forest models were trained and tested using each feature alone as a predictor. As shown in Supplementary Table S10, none of the features alone achieved as high an AUROC as the final model. Family income achieved an AUROC of 0.64 (95% CI 0.59–0.68). Each feature alone could achieve a high accuracy, but this was driven by high specificity with relatively poor sensitivity for detecting those with low cognitive ability.

## Discussion

We have shown by evaluating a variety of machine learning algorithms in a large population-based cohort that a RF model based on 15 features, all of which have potential to be collected in the perinatal period, achieved an AUROC of 0.77 for predicting low cognitive ability at age 5. At a decision threshold of 0.5, the model could correctly predict the cognitive outcome of 87% of infants, however this was largely driven by a high specificity and its ability to detect the majority of children with normal cognitive ability. When the alternative cut off of 1 SD was used, an AUROC of 0.70 was achieved using 31 features in a RF model. Model performance was similar to that reported by Camargo et al., who developed a LR model with an AUROC of 0.75, to predict low IQ (defined by a *z*-score greater than 1 standard deviation below the mean) at age 6. Their model, however, included predictors that could not be measured until the infant was 12-months old.^16^ No other similar predictive models were identified in the literature for comparison, and to our knowledge, this is the first study to examine of potential of machine learning for the prediction of low cognitive ability in childhood.

It is difficult to make direct comparisons of model performance with other screening tools due to differences in cohort characteristics, cognitive tests, and cut-off scores used to determine LCA. The available literature examining the performance of the Ages and Stages Questionnaire (ASQ) suggests very similar performance. The ASQ is one of the most widely used parent-reported screening tools and is currently recommended for early screening of developmental delay at 2 years of age in many countries, including by the American Academy of Pediatrics. The ASQ at 24 and 36 months have reported AUROCs of 0.64 and 0.78 respectively, for predicting low IQ at age 5, defined as an IQ < 1 SD below the cohort mean and an IQ < 85 in the respective studies.^33,34^ A study examining the performance of the ASQ performed between 8–30 months for predicting low IQ in the early years of schooling reported similarly low sensitivities ranging from 28–50%, with specificities ranging from 79–96% across a range of cut-offs.^35^ Our data suggest that similar prediction can be made at birth allowing early intervention in the most high risk cases.

The statistical approach used in this study was designed to optimise prediction, not investigate causal relationships. However, it is notable that among the ten most important predictors, six (alcohol intake, social class, education, income, bedrooms in the home, and maternal age) are inherently associated with socioeconomic status.^36,37^ This is in keeping with a wealth of literature, both interventional and observational, which has consistently shown that the socioeconomic environment an infant is born into is one of the strongest predictors of cognitive outcomes in childhood.^38–42^ The findings of this study would suggest that these features, while all representative of socioeconomic status, may contribute to risk in different ways as their cumulative effects better predicted the outcome than the effect of any single feature.

Previous research has shown that children exposed to multiple early risk factors represent a more vulnerable subgroup for adverse childhood outcomes. The more risk factors a child is exposed to the worse these outcomes tend to be.^43^ The most commonly used statistical approach to combining risk factors for the prediction of cognitive outcomes has been to combine them in an additive fashion, with the main effect of each factor accounted for.^15,16^ However, we know that risk factor effects are not necessarily the same for all children and effect modification and interaction does exist.^44^ The effect of one risk factor may be accentuated or diminished by another exposure. For example, the co-occurrence of brain injury due to preterm birth, a biological risk factor, and socioeconomic disadvantage, a social risk factor, have synergistic adverse effects on neurodevelopmental outcomes.^45^ An advantage of the ML approach used in this study is that the algorithms can combine features in interactive relationships without the need for prior specification.

Our study has many limitations to consider. There is much debate about the merits and limitations of standardised cognitive assessments. Both the ICD-11 and the Diagnostic and Statistical Manual of Mental Disorders 5th Edition (DSM-V) now include the impact on adaptive functioning as a diagnostic criteria for IDDs, reflecting a move away from using standardised testing alone.^24,46^ The purpose of this study was not to develop a diagnostic model, but a prognostic model that could identify high-risk children whose cognitive outcome could adversely impact other aspects of life, such as educational attainment and mental health.^47,48^ In this regard, the use of a binary cut-off is justified, although there is no consensus on the most appropriate one. In this study cut offs of 1 and 1.5 SD were examined.

Cognitive ability was directly measured using standardised assessments, however children taking the naming vocabulary test, for whom English is not their native language, may be disadvantaged, with apparent poor performance. A more appropriate measure of cognitive ability in children from multilingual backgrounds may include total vocabulary across their languages, or novel language-independent cognitive assessments.^49,50^ Children in GUI did not complete all BAS subtests and PCA was therefore used to calculate a GCA score. This may not be acceptable for a formal diagnosis of an IDD, however, a cited advantage of the BAS is that not all tests in the battery are required to assess performance and tests are individually interpretable.^21^ While there is overlap in the mental processes used in different subtests, the two subtests chosen in the GUI study largely measure verbal and non-verbal ability, and are not primarily intended to measure numerical ability, spatial ability, perceptual speed, or memory. Performing the full battery of BAS subtests would provide a more robust assessment of cognitive ability, but for large population-based cohort studies may not be feasible.^21,51^

There is now a substantial body of evidence to suggest that early interventions are most effective when started in the first year of life.^11–13^ For this reason, our study focussed on features which could be collected in the perinatal period at a population level. However, this approach may come at the cost of reduced predictive performance as detailed information on the child’s early home and school environment were not included in the model. Information on the number of books in the home and the time spent on learning activities at age 3 were included in feature set C but predictive performance was not significantly better than with perinatal features alone. This likely reflects the fact that the early learning environment is intimately intertwined with the socioeconomic background of the family.

While all 15 features in the final model could be collected in the perinatal period, in the GUI study they were collected at infant age 9-months. Validation of the model in a cohort with data collection in the perinatal period is required, but this is challenging. There are more than 110 birth cohorts in Europe, however, there are no consensus guidelines on measurements, scales, data sources, or timing.^52^ This leads to significant challenges with merging and harmonising datasets which could provide large pools of data for validation. Work in this area is ongoing.^53^

This study included a large sample size, and a careful sampling strategy was employed in recruitment of the cohort.^20^ Attrition in the GUI study was relatively low and the sample included in this study who completed cognitive assessments at age 5 represented 79.6% of the original cohort recruited at 9-months. However, attrition was higher among those from disadvantaged backgrounds, who are also more likely to have poor cognitive outcomes.^54^ Ideally, the model should be validated using population-based registry data which is not currently collected in Ireland.

Extensive validation is required to ensure wider generalisability of predictive models. If the relationship between predictor and outcome changes, model performance will be affected. For example, in Ireland only 35% of babies receive any breastmilk at 3 months, and this is significantly associated with socioeconomic status (SES).^55^ In our model breastfeeding may be a surrogate marker of SES, a relationship which may not exist in other countries where there are very high rates of breastfeeding.

An important challenge with ML models is explainability, the concept that the prediction a model makes can be explained in an acceptable way on a human level. In this study PDP plots were used to help understand how the model was making predictions. However, these must be interpreted with caution. For example, the PDP plot examining the relationship between PCG alcohol intake and risk of low cognitive ability in the child would suggest that those with moderate alcohol consumption have the lowest risk, while those with no alcohol or very high alcohol consumption have the highest risk. This U-shaped relationship curve between alcohol consumption and health outcomes has been described previously in the literature.^56^ In our study, among those who reported English was not their native language 41.4% reported no alcohol intake, compared to only 12% of those for whom English was the native language. Therefore, it is plausible that the increased risk seen for those who reported no alcohol consumption is due to confounding, and the relationship is actually being driven by immigration, cultural, religious, language, or socioeconomic factors.

Finally the individual, health-system, and resource implications of a risk-based approach must be considered. In its current iteration this model may not be suitable for use at a population level. The sensitivity is too low and the resource implications of the high false positive rate, when the decision threshold is lowered, is too great. However, it is a first step towards enabling an early personalised, prediction, which could assist decisions on further intervention. This is not without consequence. Labelling a child so early in life could have detrimental effects on both child and family. In addition to adverse impacts on the child’s self-concept, it can perpetuate a self-fulfilling prophecy due to effects on parent and teacher expectations.^57,58^ Using a label based largely on social factors, often rooted in inequities generated by social and political policy, may shift the blame of societal failures onto the individual child and family. Any risk stratification must come from a position of support, environmental enrichment, and education.

In conclusion, the current practice of waiting for overt signs of developmental delay before intervention goes against a large body of developmental literature. In this study, it was possible to develop a model, using 15 simple features available at birth and readily incorporated into an electronic health record, that correctly predicted the cognitive ability of 87% of children at age 5. Whilst further improvements in predictive performance would be required to use this as a population level screening tool, it provides a strong basis for further research. New methods of direct assessment of early cognitive function are lacking. Neurophysiological measures such as electroencephalography and eye-tracking show some promise.^42,59^ Combined with targeted direct assessment, this model could, in the future, form the foundation for an early targeted screening protocol similar to that now being widely implemented for the early diagnosis of cerebral palsy.^60,61^ Improved capacity to collect large volumes of rich data and to apply novel statistical methods, particularly suited to prediction, provides an opportunity for researchers and clinicians to investigate alternative approaches.

### Supplementary information

Supplementary material

## Data Availability

The anonymised Growing Up in Ireland data from the Infant Cohort is available for request for bona fide research purposes through the Irish Social Science Data Archive https://www.ucd.ie/issda/data/growingupinirelandgui/.
