# Supplementary material for: Predicting low cognitive ability at age 5 years using perinatal data and machine learning
Source: Pediatr Res. 2024 Jan 4;95(6):1634–43. doi: 10.1038/s41390-023-02914-6 (PMC11126385; doi:10.1038/s41390-023-02914-6)
Supplement: Supplementary file 1 — Supplementary material [file 41390_2023_2914_MOESM1_ESM.pdf]

Supplementary material

Text

Evaluation Metrics

Sensitivity = $\frac{TP}{TP+FN}$

Specificity = $\frac{TN}{TN+FP}$

$$\frac{TN}{TN+FP}$$

Accuracy = $\frac{TP+TN}{TP+FP+TN+FN}$

Supplementary Figure Legends

Figure S1. Flowchart of study population in Growing Up in Ireland (GUI) study
This figure shows the number of participants at each wave of the GUI study. Shown in the boxes to the right are the number invited to participate at each wave, and the reasons for non-participation.

Figure S2. Framework for feature selection
This figure demonstrates the theoretical framework used to identify potential features in the Growing Up in Ireland dataset. All features considered in the feature selection process are shown.

Figure S3. Receiver operating characteristic curves of Models 1-3
This figure shows the receiver operating characteristic curves for prediction of low cognitive ability at age 5 for Random Forest Model 1 (8 features), Model 2 (15 features), Model 3 (23 features). Model 3 achieved the highest area under the curve (AUC) of 0.776.

Figure S4. Overview of modelling process for cognitive ability ≥ 1SD below mean
This figure shows the modelling process and numbers involved at each stage of modelling using an alternative cut off point of a general cognitive ability score ≥ 1SD below mean. Those below the cut-off were categorised as below average cognitive ability (BACA).

Figure S1. Flowchart of study population in Growing Up in Ireland (GUI) study

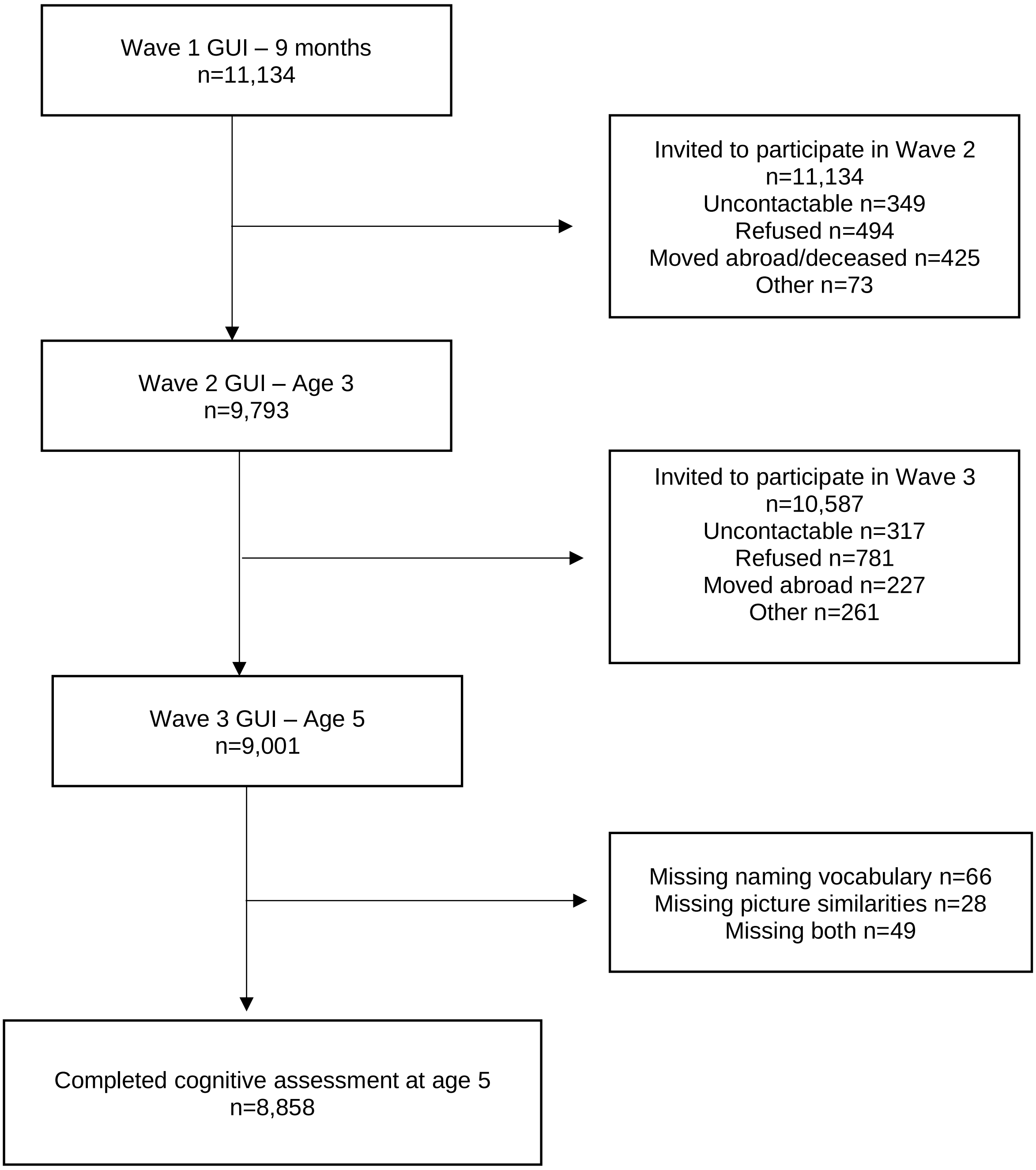

Figure S2. Framework for feature selection

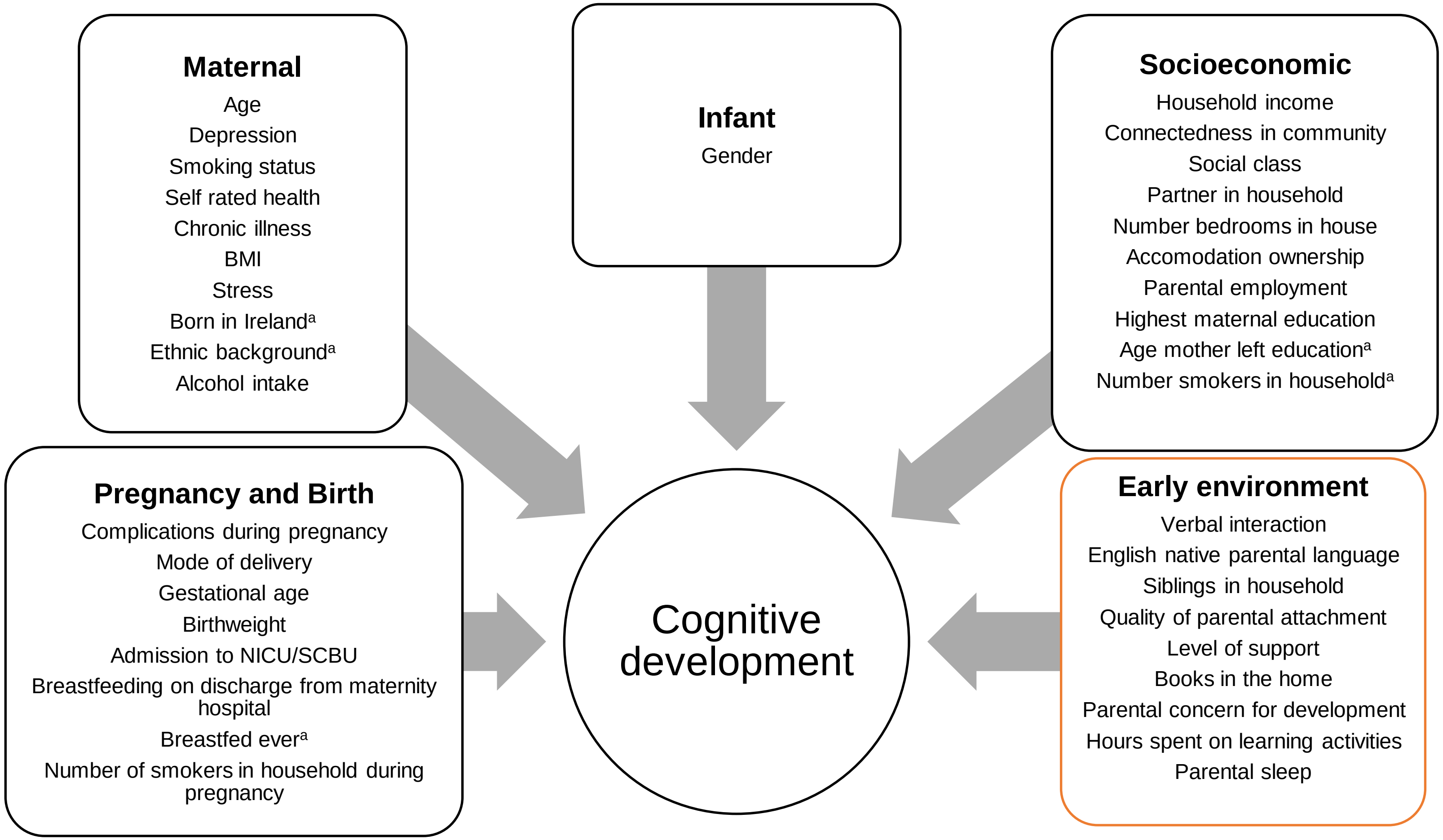

[a]Feature removed after examining correlations for redundancy

Figure S3. Receiver operating characteristic curves of Models 1-3

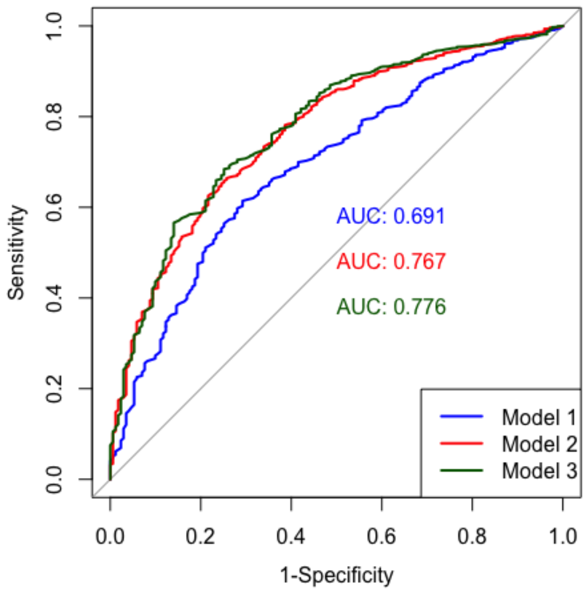

Table S1. Subtests of the British Ability Scales Second Edition Early Years Battery[21,61]

Name of Scale	Ability being tested
Naming vocabulary	Expressive verbal knowledge
Picture similarities	Non-verbal reasoning
Verbal comprehension	Receptive verbal knowledge
Early number concepts	Quantitative knowledge reasoning
Block building	Perceptual motor ability
Pattern construction	Spatial problem solving
Copying	Perceptual motor ability
Matching letter-like forms	Visual perceptual matching
Recall of digits forward	Short term auditory memory
Recall of objects	Short and intermediate term visual verbal memory
Recognition of pictures	Short term visual memory

[a] The total number of subtests administered is dependent on the age of the child being tested and ranges between 8-11 for children age 3 years 6 months – 5 years 11 months. The core subtests produce a measure of verbal ability (naming vocabulary and verbal comprehension), non-verbal ability (picture similarities and block building), and spatial ability (pattern construction and copying).

Table S2. Description of features considered in feature selection process

Feature number	Feature	Details	Type
	**Pregnancy and birth**		
1	Hypertension	Yes, No	Factor
2	Pre-eclampsia	Yes, No	Factor
3	Urinary tract infection	Yes, No	Factor
4	Persistent nausea and vomiting	Yes, No	Factor
5	Gestational diabetes (controlled by diet)	Yes, No	Factor
6	Gestational diabetes (controlled by insulin)	Yes, No	Factor
7	Bleeding late pregnancy	Yes, No	Factor
8	Vaginal infection	Yes, No	Factor
9	Intrauterine growth restriction	Yes, No	Factor
10	Mode delivery	Normal, forceps/suction assisted, planned c-section, emergency c-section	Factor
11	Gestational age	Gestational age in weeks	Numeric
12	Birthweight category	<1500g, 1500-2000g, 2500-4500g, >4500g	Ordinal. Treated as numeric in model.
13	Required neonatal intensive care unit (NICU) or special care baby unit (SCBU)	Yes, No	Factor
14	Breastfed on discharge from hospital	Yes, No	Factor
15	Number of people who smoked in household during pregnancy	Numeric (response of 5 = 5 or more)	Numeric
	**Maternal**		
16	Age	Age of PCG when infant age 9 months (response 40 = 40 or more years)	Numeric
17	Body mass index	Weight in kg/height m^2 derived from measured data	Numeric
18	Self-rated health	Excellent, very good, good, fair, poor	Ordinal. Treated as numeric in model
19	Depression score	Centre for Epidemiological Studies Depression Scale (short version)	Numeric
20	Chronic illness (includes physical, mental, disability)	Yes, No	Factor
21	Current smoker (daily or occasionally)	Yes, No	Factor

22	Alcohol intake	Never (1), less than once a month (2), 1-2/month (3), 1-2 times/week (4), 3-4 times/week (5), 5-6 times/week (6), daily (7)	Ordinal. Treated as numeric in model.
23	Stress	Parental stress scale	Numeric
24	Born in Ireland	Yes, No	Factor
25	Ethnic background	Irish, any other white background, African or any other Black background, Chinese or any other Asian background, Other	Factor
26	Infant		
27	Infant gender	Male, Female	Factor
	**Socioeconomic**		
28	Number of bedrooms	Number of separate bedrooms in accommodation	Numeric
29	Number in household who currently smoke	Number who currently smoke (response of 6 = 6 or more)	Numeric
30	Accommodation ownership	Owner occupied (with or without mortgage), being purchased from a local authority, rented from a local authority, rented from a voluntary body, rented from private landlord, living with and paying rent to parents, occupied free of rent	Factor
31	PCG employment status	Employee, self-employed, farmer, student full-time, state training scheme, unemployed actively looking for job, long-term sickness or disability, home duties, retired, other	Factor
32	Household equivalised income	Calculated as disposable household income divided by equivalised household size.	Numeric
33	PCG highest education	Primary or less, lower secondary, upper secondary, technical/Vocational, non-degree, degree or equivalent, post degree	Ordinal. Treated as numeric in model.

		cert/diploma, postgrad masters or PhD	
34	Family's social class	Highest social class category of both parents in family (where relevant) based on their occupation. Professional workers (1), managerial and technical (2), non-manual (3), skilled manual (4), semi-skilled (5), unskilled (6), all others gainfully occupied (7), never worked at all (8)	Ordinal. Treated as numeric in model.
35	Connectedness in community	As a family are settled and part of community – Strongly agree, Agree, Disagree, Strongly disagree	Ordinal. Treated as numeric in model
	**Early environment**		
36	Siblings	Brothers or sisters living in household. Yes, No.	Factor
37	Parental sleep	Average hours of sleep nightly. Numeric (response of 3 = 3 or less)	Numeric
38	Verbal interaction with baby	Parent talks to baby when busy doing things - Never, rarely, sometimes, often, always	Ordinal. Treated as numeric in model.
39	Native language of primary care giver (PCG) is English	Yes, No	Factor
40	Quality of parental attachment (PCG)	The Quality of Attachment subscale from the Maternal Postnatal Attachment Scale	Numeric
41	Level of PCG support	I get enough help, I don't get enough help, I don't get any help at all, I don't need any help, Family not living in the country	Factor
42	Books in the home	How many children's books does child have access to in the home now, including any library books – None, <10, 10-20, 21-30, >30	Factor

43	Reading	Days per week someone reads to child in the home	Numeric
44	Alphabet	Days per week someone does alphabet with child in the home	Numeric
45	Numeracy	Days per week someone does numbers or counting with child in the home	Numeric
46	Music	Days per week someone does songs, poems, or nursery rhymes with child in the home	Numeric
47	Games	Days per week someone play games (board games, jigsaws, card games etc) with child in the home	Numeric
48	Art	Days per week someone paints, draws, colours, or plays with play-doh at home with child in the home	Numeric
49	Active play	Days per week someone plays active games with child (e.g. football)	Numeric
50	Hours spent on learning activities	Sum of items 51-57	Numeric

All features (excluding features 42-50*) were measured at the 9-month questionnaire which was a self-complete questionnaire completed by the child's primary caregiver which in 99.7% of cases was the child's mother. *Features 42-50 were measured at the 3-year questionnaire.

Table S3. Feature sets used in Recursive Feature Elimination process

	Feature set 1	Feature set 2	Feature set 3
Pregnancy and Birth	Gestational age*<br>Admission to NICU/SCBU	Hypertension<br>Pre-eclampsia<br>Urinary tract infection<br>Persistent nausea/vomiting<br>Gestational diabetes (controlled by diet)<br>Gestational diabetes (controlled by insulin)<br>Bleeding late pregnancy<br>Vaginal infection<br>Intrauterine growth restriction<br>Mode of delivery<br>Gestational age*<br>Birthweight category*<br>Admission to NICU[a]/SCBU[b]<br>Number in household smoking during pregnancy*<br>Breastfed on discharge from maternity hospital	Hypertension<br>Pre-eclampsia<br>Urinary tract infection<br>Persistent nausea/vomiting<br>Gestational diabetes(controlled by diet)*<br>Gestational diabetes (controlled by insulin)<br>Bleeding late pregnancy<br>Vaginal infection<br>Intrauterine growth restriction<br>Mode of delivery<br>Gestational age*<br>Birthweight category*<br>Admission to NICU/SCBU<br>Number in household smoking during pregnancy*<br>Breastfed on discharge from maternity hospital
Maternal	BMI*<br>Highest level of education*<br>Depression*<br>Alcohol intake*<br>Age*	BMI*<br>Highest level of education*<br>Depression*<br>Alcohol intake*<br>Age*<br>Self-rated health*<br>Chronic illness<br>Smoking status	BMI*<br>Highest level of education*<br>Depression*<br>Alcohol intake*<br>Age*<br>Self-rated health*<br>Chronic illness<br>Smoking status<br>Stress*
Infant	Gender	Gender	Gender
Socioeconomic	Household social class*<br>Household income*<br>Partner in household	Household social class*<br>Household income*<br>Maternal employment status<br>Number of bedrooms*	Household social class*<br>Household income*<br>Maternal employment status*<br>Number of bedrooms*

		Accommodation ownership*<br>Connectedness in community*<br>Partner in household	Accommodation ownership*<br>Connectedness in community*<br>Partner in household
Early environment		Siblings<br>English is maternal native language*	Siblings<br>English is maternal native language*<br>Books in the home*<br>Hours spent on learning activities*<br>Verbal interaction*<br>Parental sleep*<br>Level of support*<br>Quality of parental attachment*

* Selected in recursive feature elimination for final model; [a]Neonatal intensive care unit; [b]Special care baby unit

Table S4. Pregnancy, maternal, infant, socioeconomic and early environment characteristics of study population

Characteristics	Total	Low n=573 (6.5%)	Other n=8,285 (93.5%)	p-value
**Pregnancy and Birth**				
**Mode of delivery**				
Normal vaginal	5,239 (59.1)	367 (64.0)	4,875 (58.8)	
Forceps/suction assisted	1,299 (14.7)	60 (10.5)	1,238 (14.9)	
Planned c-section	1,151 (13.0)	61 (10.6)	1,090 (13.2)	
Emergency c-section	1,169 (13.2)	85 (14.8)	1,082 (13.1)	0.003[b]
Preeclampsia	620 (7.0)	35 (6.1)	585 (7.1)	0.436[b]
Gestational diabetes (controlled by diet)	195 (2.2)	16 (2.8)	179 (2.2)	0.396[b]
Gestational diabetes (controlled by insulin)	83 (0.9)	3 (0.5)	80 (1.0)	0.373[e]
IUGR[a]	179 (2.0)	12 (2.1)	167 (2.0)	1.000[b]
Admission to NICU/SCBU	1,217 (13.7)	88 (15.4)	1,129 (13.6)	0.271[b]
Gestational age – mean (sd)	39.5 (2.1)	39.3 (2.4)	39.5 (2.0)	0.035[c]
**Birthweight**				
<1,500g	67 (0.8)	6 (1.0)	61 (0.7)	
1500g-2500g	401 (4.5)	38 (6.6)	362 (4.4)	
2500g-4500g	8,178 (92.3)	509 (88.8)	7,670 (92.6)	
>4500g	212 (2.4)	20 (3.5)	192 (2.3)	0.0138[e]
Breastfed on discharge - yes	4,131 (46.6)	300 (52.4)	4,427 (53.4)	0.648[b]
Smoker in hsd smoking during pregnancy - yes	2,801 (31.6)	225 (39.3)	2,576 (31.1)	<0.001[b]
**Maternal**				
Age – mean (sd)	32.0 (5.2)	30.6 (5.7)	32.1 (5.1)	<0.001[c]
BMI – mean (sd)	25.5 (4.7)	25.9 (5.0)	25.5 (4.7)	0.072[c]
**Self-rated health**				
Excellent	2,798 (31.6)	159 (27.7)	2,639 (31.9)	
Very good	3,505 (39.6)	223 (38.9)	3,282 (39.6)	
Good	2,020 (22.8)	150 (26.2)	1,870 (22.6)	
Fair	475 (5.4)	35 (6.1)	440 (5.3)	
Poor	60 (0.6)	6 (1.0)	54 (0.7)	0.097[b]
Current smoker - yes	2,028 (22.9)	174 (30.4)	1,854 (22.4)	<0.001[b]
**Alcohol intake**				
Never	1,457 (16.4)	192 (33.5)	1,265 (15.3)	
<1/month	2,409 (27.2)	178 (31.1)	2,231 (26.9)	
1-2/month	2,398 (27.1)	119 (20.8)	2,279 (27.5)	
1-2/week	2,176 (24.6)	73 (12.7)	2,103 (25.4)	
3-4/week	353 (4.0)	10 (1.7)	343 (4.1)	
5-6/week	49 (0.6)	<5	48 (0.6)	
Daily	16 (0.2)	<5	16 (0.2)	<0.001[e]
Maternal depression – median (IQR)	1 (3)	1 (3)	1 (3)	0.0384[d]
Chronic illness - yes	1,019 (11.5)	55 (9.6)	964 (11.6)	0.159[b]
**Highest level of education**				
Primary or less	186 (2.1)	43 (7.5)	143 (1.7)	

Lower secondary	739 (8.3)	77 (13.4)	662 (8.0)	
Upper secondary	1,608 (18.2)	131 (22.9)	1,477 (17.8)	
Technical/vocational	1,194 (13.5)	114 (19.9)	1,080 (13.0)	
Non Degree	1,787 (20.2)	74 (12.9)	1,713 (20.7)	
Degree or equivalent	1,617 (18.3)	68 (11.9)	1,549 (18.7)	
Post degree cert/diploma	1,047 (11.8)	36 (6.3)	1,011 (12.2)	
Post degree masters or PhD	680 (7.7)	30 (5.2)	650 (7.8)	<0.001[b]
**Infant**				
Infant gender - male	4,479 (50.6)	347 (60.6)	4,132 (49.9)	<0.001[b]
**Socioeconomic**				
Household income – median (IQR)	48,000 (33,600)	31,200 (24,963)	48,000 (33,330)	<0.001[d]
**Household social class**				
Professional workers	1,711 (19.3)	52 (9.1)	1,659 (20.0)	
Managerial and technical	2,863 (32.3)	109 (19.0)	2,754 (33.2)	
Non-manual	1,472 (16.6)	85 (14.8)	1,387 (16.7)	
Skilled manual	1,168 (13.2)	122 (21.3)	1,046 (12.6)	
Semi-skilled	638 (7.2)	72 (12.6)	566 (6.8)	
Unskilled	119 (1.3)	15 (2.6)	104 (1.3)	
All others gainfully occupied	37 (0.4)	8 (1.4)	29 (0.4)	
Never worked	850 (9.6)	110 (19.2)	740 (8.9)	<0.001[b]
**Accommodation occupancy**				
Owner occupied	6,315 (71.3)	223 (38.9)	6,091 (73.5)	
Local authority housing	623 (7.0)	85 (14.8)	538 (6.5)	
Private rental	1,622 (18.3)	249 (43.5)	1,374 (16.6)	
Living with parents	146 (1.6)	8 (1.4)	138 (1.7)	
Occupied rent free	152 (1.7)	8 (1.4)	144 (1.7)	<0.001[b]
Number of bedrooms – mean (sd)	3.5 (0.9)	3.1 (0.9)	3.5 (0.9)	<0.001[c]
**Connectedness in community**				
Strongly agree	3,777 (42.6)	186 (32.5)	3,591 (43.3)	
Agree	4,292 (48.5)	320 (55.8)	3,972 (47.9)	
Disagree	618 (7.0)	54 (9.4)	564 (6.8)	
Strongly disagree	171 (1.9)	13 (2.3)	158 (1.9)	<0.001[b]
**Early environment**				
Siblings - yes	5,410 (61.1)	355 (62.0)	5,055 (61.0)	0.687[b]
**Books in home**				
None	29 (0.3)	6 (1.0)	23 (0.3)	
<10	585 (6.6)	111 (19.4)	474 (5.7)	
10-20	1,626 (18.4)	169 (29.5)	1,457 (17.6)	
21-30	1,562 (17.6)	105 (18.3)	1,457 (17.6)	
>30	5,056 (57.1)	182 (31.8)	4,874 (58.8)	<0.001[b]
Hours on learning activities – mean (sd)	34.2 (8.5)	31.8 (9.9)	34.4 (8.3)	<0.001[c]
**Level of support**				
Get enough	6,446 (72.8)	344 (60.0)	6,102 (73.7)	
Don’t get enough	856 (9.7)	49 (8.6)	807 (9.7)	
Don’t get any	482 (5.4)	36 (6.3)	446 (5.4)	
Don’t need any	458 (5.2)	51 (8.9)	407 (4.9)	
Family not in country	616 (7.0)	93 (16.2)	523 (6.3)	<0.001[b]

Quality of parental attachment – median (IQR)	44 (4)	44 (4)	44 (4)	0.529[d]
Parental sleep hours/night – median (IQR)	11 (2)	10 (1)	11 (2)	<0.001[d]
English maternal native language – yes	7,512 (84.8)	309 (53.9)	7,203 (86.9)	<0.001[b]
**Verbal interaction – talk to baby while busy doing things**				
Never	201 (2.3)	16 (2.8)	185 (2.2)	
Rarely	33 (0.4)	<5	30 (0.4)	
Sometimes	658 (7.4)	50 (8.7)	608 (7.3)	
Often	2,130 (24.0)	143 (25.0)	1,987 (24.0)	
Always	5,836 (65.9)	361 (63.0)	5,475 (66.1)	0.385[e]
Partner in household	7,929 (89.5)	491 (85.7)	7,438 (89.8)	0.003[b]

[a]Intrauterine growth restriction; [b]Pearson's Chi-squared test; [c]Welch two sample t-test; [d]Wilcoxan rank sum test ; [e]Fisher's Exact Test (p-value simulated where cell number too small);

Table S5. Average accuracy of machine learning algorithms across 10-fold cross validation on training data calculated at a decision threshold of 0.5

	Model 1 (8 features)	Model 2 (15 features)	Model 3 (24 features)
Random forest	0.85 (0.02)	0.87 (0.02)	0.87 (0.03)
Logistic regression	0.70 (0.01)	0.75 (0.01)	0.76 (0.01)
Support vector machine	0.78 (0.02)	0.81 (0.03)	0.80 (0.03)

Table S6. Performance of Model 2 across different decision thresholds on independent test set

Decision threshold	Sensitivity	Specificity	Accuracy
0.50	0.40	0.91	0.87
0.45	0.47	0.87	0.84
0.40	0.55	0.82	0.80
0.35	0.61	0.76	0.75
0.30	0.69	0.70	0.70
0.28[a]	0.72	0.66	0.68

[a] Optimal cut point chosen using Youden's J statistic where the optimal cut-off is the threshold that maximises the distance to the diagonal line)

Table S7. Contingency table showing how model would perform across 60,000 births at a decision threshold of 0.5

		Standardised cognitive test age 5		
		Low	'Normal'	
Model predictions	Low cognitive ability	1,560	5,610	7,170
	'Normal cognitive ability'	2,340	50,490	52,830
		3,900	56,100	60,000

Supplementary analysis using a cognitive ability score cut-off of >1 standard deviation below the mean to define low cognitive ability.

Figure S4. Overview of modelling process for cognitive ability > 1SD below mean (Below average cognitive ability (BACA)

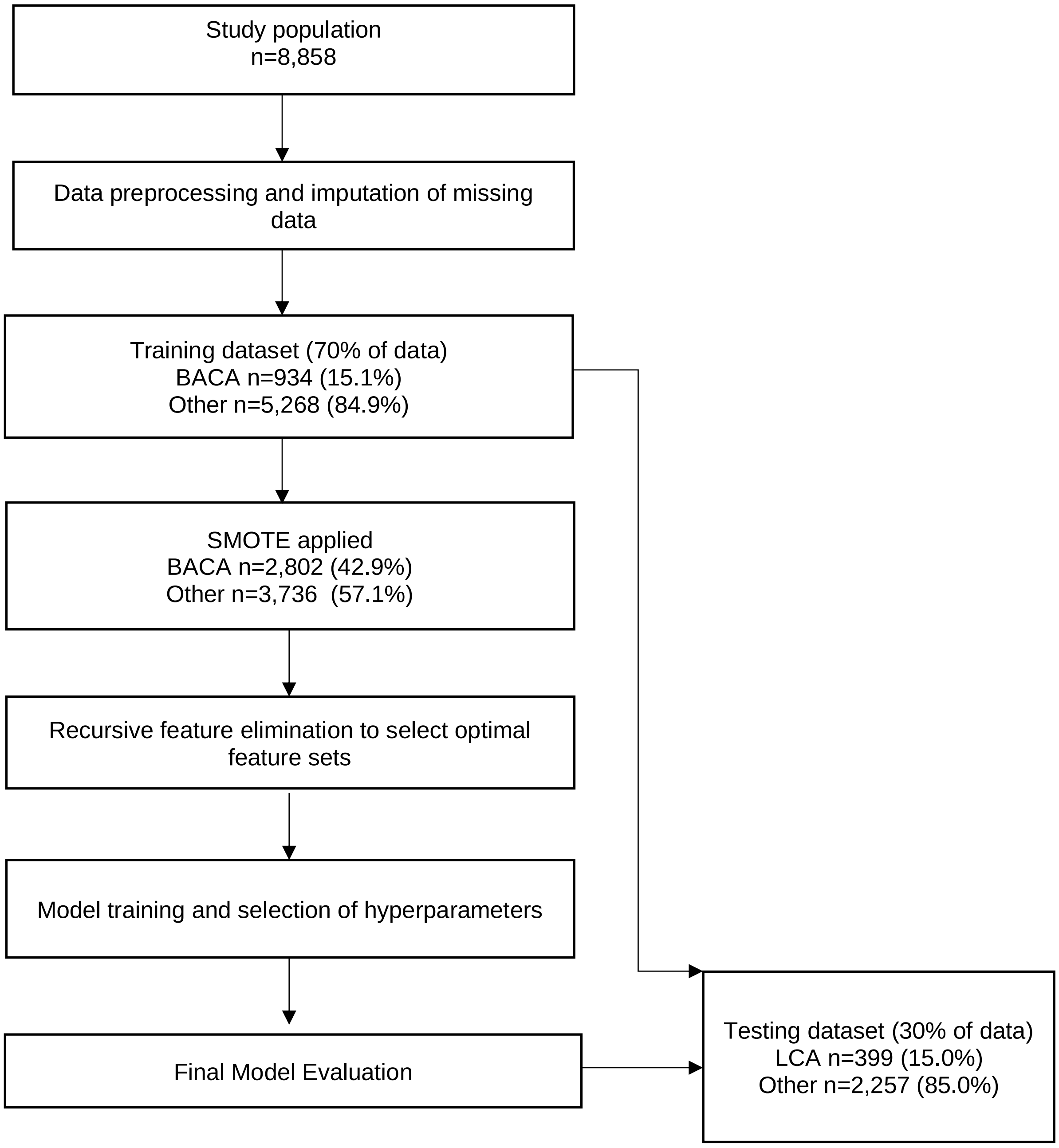

Table S8. Accuracy of different machine learning algorithms on 10-fold cross-validation for predicting cognitive ability > 1 standard deviation below mean at age 5

	Model 1<br>(9 features)	Model 2<br>(18 features)	Model 3<br>(31 features)
Accuracy[a]			
Random forest	0.86 (0.01)	0.87 (0.01)	0.88 (0.01)
Logistic regression	0.67 (0.01)	0.71 (0.01)	0.73 (0.01)
SVM	0.77 (0.01)	0.81 (0.02)	0.82 (0.02)

[a] Calculated at a decision threshold of 0.5

Table S9. Performance metrics of final models tested on independent test set for predicting cognitive ability > 1 standard deviation below mean at age 5

	Model 1<br>(9 features)	Model 2<br>(18 features)	Model 3<br>(31 features)
Accuracy[a]	0.82 (0.80-0.83)	0.82 (0.81-0.84)	0.82 (0.81-0.84)
Sensitivity[a]	0.22	0.29	0.35
Specificity[a]	0.92	0.92	0.91
AUROC[b]<br>(95% confidence interval)	0.63 (0.60-0.66)	0.68 (0.65-0.71)	0.70 (0.66-0.72)
Positive predictive value[a]	0.33	0.39	0.40
Negative predictive value[a]	0.87	0.88	0.89

[a] Calculated at a decision threshold of 0.5, [b] Area under receiver operating characteristic curve

Table S10. Performance metrics of random forest models trained with the five most important socioeconomic features alone.

Feature	AUROC (95% CI)	Accuracy[a]	Sensitivity[a]	Specificity[a]
Household social class	0.56 (0.52-0.59)	0.94 (0.93-0.94)	0.00	1.00
PCG Highest education	0.55 (0.52-0.58)	0.92 (0.91-0.93)	0.06	0.98
Household income	0.64 (0.59-0.68)	0.80 (0.78-0.81)	0.33	0.83
Number of bedrooms in home	0.52 (0.50-0.54)	0.94 (0.93-0.94)	0.00	1.00
PCG alcohol intake	0.62 (0.58-0.66)	0.81 (0.79-0.82)	0.28	0.84

[a] Calculated at a decision threshold of 0.5
